# Herbal Decoction Divya-Peedantak-Kwath Alleviates Allodynia and Hyperalgesia in Mice Model of Chemotherapy-Induced Peripheral Neuropathy via Modulation in Cytokine Response

**DOI:** 10.3389/fphar.2020.566490

**Published:** 2020-10-30

**Authors:** Acharya Balkrishna, Sachin S. Sakat, Shadrak Karumuri, Hoshiyar Singh, Meenu Tomer, Ajay Kumar, Niti Sharma, Pradeep Nain, Swati Haldar, Anurag Varshney

**Affiliations:** ^1^ Drug Discovery and Development Division, Patanjali Research Institute, Haridwar, India; ^2^ Department of Allied and Applied Sciences, University of Patanjali, Haridwar, India

**Keywords:** chemotherapy-induced peripheral neuropathy, herbal decoction, cytokine modulation, allodynia, hyperalgesia, mouse model

## Abstract

The widely used cancer treatment, chemotherapy, causes severe long-term neuropathic pain in 30–40% cases, the condition clinically known as chemotherapy-induced peripheral neuropathy (CIPN). Approved conventional analgesics are sometimes ineffective, while others like opioids have undesirable side effects like addiction, seizures, and respiratory malfunctioning. Tricyclic antidepressants and anticonvulsants, although exhibit anti-allodynic effects in neuropathy, also have unpleasant side effects. Thus, alternative medicines are being explored for CIPN treatment. Despite scattered reports on different extracts from different plants having potential anti-allodynic effects against CIPN, no established medicine or formulation of herbal origin exists. In this study, efficacy of an herbal decoction, formulated based on ancient medicinal principles and protocols for treating neuropathic pain, Divya-Peedantak-Kwath (DPK), has been evaluated in a paclitaxel (PTX)-induced peripheral neuropathic mouse model. We observed that DPK has prominent anti-allodynic and anti-hyperalgesic effects and acts as a nociceptive modulator for CIPN. With exhibited antioxidative effects, DPK restored the redox potential of the sciatic nerves to the normal. On histopathological evaluation, DPK prevented the PTX-induced lesions in the sciatic nerve, in a dose-dependent manner. It also prevented inflammation by modulating the levels of pro-inflammatory cytokines involved in CIPN pathogenesis. Our observations evinced that DPK can alleviate CIPN by attenuating oxidative stress and concomitant neuroinflammation through immune modulation.

## Introduction

Chemotherapy-induced peripheral neuropathy (CIPN), one of the most common side effects of chemotherapeutic agents, has symptoms like pain, allodynia, loss of sensation, paresthesia, numbness, tingling, and gait disturbances (Miltenburg and Boogerd, 2014; Heuvel et al., 2017). CIPN quite often leads to reduction in the dose of the chemotherapeutic agent being administered or, in the worst case, discontinuation of the treatment altogether. This not only affects the quality of life but also puts the survival of the patient at stake (Miltenburg and Boogerd, 2014). Particularly, with extension in cancer patient survival times due to newer therapies, CIPN is becoming more prevalent. A staggering 68% of CIPN prevalence, in the first month of chemotherapy that reduces only to 60 and 30% in the subsequent three and six months, respectively, is indeed alarming. These figures are based on a review of data from 4,179 patients across 31 studies, according to PRISMA (Preferred Reporting Items for Systematic reviews and Meta-Analyses) guidelines. The dataset analyzed in this review included reports from CIPN cases due to treatments with oxaliplatin, bortezomib, placlitaxel, taxane, cisplatin, vincristine, thalidomide, platinum, carboplatin, docetaxel, and proteasome inhibitor (Seretny et al., 2014). As high as 80% CIPN prevalence for as long as 6 months to 2 years has been reported in patients receiving taxanes and oxaliplatin therapy (Loprinzi et al., 2011; Briani et al., 2014). Unfortunately, available treatment options for CIPN are limited, often ineffective or with undesirable side effects. Moreover, underlying pathophysiology of CIPN is still somewhat less understood. Considering CIPN as neuropathic pain due to axonal degeneration, relevant pharmacologic treatments, involving tricyclic antidepressants and anticonvulsants, are administered. However, these agents showed suboptimal efficacy against CIPN and, not to mention, have unacceptable aftereffects (Rao et al., 2007; Rao et al., 2008; Fukuda et al., 2017). This implicates that the disease mechanism behind CIPN is different from the one responsible for typical neuropathic pain. Based on the modestly positive outcome of randomized control clinical trial, the American Society of Clinical Oncology (ASCO) has recommended the use of duloxetine against CIPN. However, duloxetine is more effective in case of central neurotoxicity, characterized by mental confusion, catatonia, and hyporeflexia that particularly occur under oxaliplatin treatment (Quintão et al., 2019). Besides, duloxetine has side effects like nausea, dry mouth, sleepiness, fatigue, constipation, loss of appetite, increased sweating, and dizziness (Hershman et al., 2014; Hou et al., 2018).

Other approved and licensed drugs for neuropathic pain also have associated side effects. Conventional analgesics like nonsteroidal anti-inflammatory drugs and opioids are ineffective clinically in attenuating neuropathic pain. Besides, opioids are addictive and cause seizures and respiratory malfunctioning. Despite their reported anti-allodynic effects, use of tricyclic antidepressants and anticonvulsants against neuropathy is limited again due to severe side effects including, blurred vision, dry mouth, constipation, weight gain or loss, low blood pressure, rashes, hives, and increased heart rate. Therefore, identification of an alternative treatment for CIPN with less or no side effects is essential. Different traditional systems of medicine, with recommendations for herbal treatments, have been used throughout the world for treating neuropathic pain. This encouraged researchers to explore plants like *Acorus calamus*, *Artemisia dracunculus*, *Aconiti tuber*, *Cannabis sativa*, *Emblica officinalis*, *Ginkgo biloba*, *Nigella sativa*, *Vochysia divergens*, *Allium sepa*, and *Allium sativum* as sources for medicine against CIPN (Garg and Adams, 2012). However, these plant sources have been evaluated individually for their potency against CIPN. To the best of our knowledge, no medicinal formulation based on the traditional treatment protocols is known yet.

In this study, we have evaluated the pharmacological effects of a water-based herbal decoction, Divya-Peedantak-Kwath (DPK), in treating CIPN. DPK contains relevant parts of 28 different medicinal plants in optimized amounts. Paclitaxel (PTX)-induced mouse peripheral neuropathic model has been employed. PTX doses used to induce neuropathy were comparable to those used in humans for chemotherapy (Thangamani et al., 2013; Kaur and Muthuraman, 2019). PTX-induced neuropathy is linked to mitochondrial dysfunction due to oxidative stress that evoked antinociceptive effects (Griffiths and Flatters, 2015; Duggett et al., 2017). Therefore, in addition to evaluating the anti-allodynic and anti-hyperalgesic properties and its capability to modulate nociception in neuropathic models through behavioral tests, antioxidant profiling of DPK-treated nerve tissues was also undertaken. Since oxidative stress is linked to axonal degeneration, histological analysis and scoring of the nerve tissue have been performed to evaluate the effect of DPK treatment in attenuating the degenerative features associated with PTX-induced neuropathy (Peters et al., 2007; Tasnim et al., 2016). In addition, the anti-inflammatory profile of DPK was evaluated using human differentiated macrophage cell lines for the measurement of pro-inflammatory cytokine release. Taken together, we report that DPK is not only a potent anti-allodynic and an anti-hyperalgesic agent but also a very efficient antioxidant that can effectively attenuate the neuroinflammation associated with PTX-induced neuropathy through cytokine modulation.

## Materials and Methods

### Chemicals and Reagents

For the present study, Divya-Peedantak-Kwath (DPK) (batch no: A-PTH037, manufactured date: July 2019) was obtained from Divya Pharmacy, A-1 Industrial Area, Haridwar, India. Culture media RPMI-1640, fetal bovine serum, and antibiotic/antimycotic mixture were obtained from Gibco, and cytokines interleukin IL-1β, IL-6, and tumor necrosis factor-α (TNF-α) ELISA kits were purchased from BD Biosciences. Lipopolysaccharide (LPS) was purchased from Sigma-Aldrich (St. Louis, MO, United States). Phorbol 12-myristate 13-acetate (PMA) was purchased from Fisher Scientific, United States. Paclitaxel injection IP (brand name—MITOTAX 100; marketed by Dr Reddy’s Laboratories Ltd., Hyderabad, India) was procured from local pharmacy; gabapentin was procured from TCI Chemical Industry Co. Ltd., Tokyo, Japan.

### High-Performance Liquid Chromatography Analysis of Divya-Peedantak-Kwath

Chemical composition of DPK was analyzed through high-performance liquid chromatography (HPLC) (Waters Corporation, United States); instrument equipped with a binary pump (1525), PDAD (2998) and an autosampler (2707). Separation was achieved using a Shodex C18-4E (5 μm, 4.6 * 250 mm) column through binary gradient elution. The two solvents, designated as A and B, were used for the analysis. Both A and B had 0.1% orthophosphoric acid. But in case of B, it was in a mixture of acetonitrile and water in 88:12 ratios, while it was in water for A. pH of both solvents was set to 2.5 with diethylamine. The gradient program for the solvent system used was 95–85% A for 0–10 min, 85% A for 10–15 min, 85–80% A for 15–20 min, 80–70% A for 20–30 min, 70–40% A for 30–40 min, 40–10% A for 40–45 min, 10–95% A for 45–46 min, and 95% A for 46–50 min, with a flow rate of 1.0 ml/min 1.0 g of the sample was suspended in 20 ml methanol: water (10:90) mixture sonicated for 30 min, centrifuged at 10,000 rpm for 5 min, and filtered through a 0.45-µm nylon filter before loading on to the column. Then, 10 µl of the test solution was used for analysis. The column temperature was maintained at 35°C. Wavelengths were set to 270 nm (for gallic acid, vanillic acid, corilagin, rutin, and ellagic acid) and 325 nm (for neochlorogenic acid and cryptochlorogenic acid). Standard compounds were purchased from Natural Remedies Pvt. Ltd, Sigma Aldrich, Bangalore, and ChemFaces, China, and were dissolved in methanol at recommended concentrations.

### Cell Culture for *In Vitro* Experiments

THP-1 cell line was obtained from the ATCC-licensed cell repository National Centre for Cell Science, Pune, India. These cells were cultured in RPMI-1640 media; supplemented with 10% heat-inactivated fetal bovine serum, in the presence of penicillin–streptomycin (100 U/ml), sodium pyruvate (1 mM), and l-glutamine (4 mM); and grown at 37°C in a CO_2_ incubator with 5% CO_2_. All experiments were done with cells between passage numbers 8 and 10.

### Evaluation of Anti-Inflammatory Activity and Cytotoxicity of Divya-Peedantak-Kwath *In Vitro*

Water-extracted DPK powder was dissolved in incomplete RPMI-1640 culture medium. THP-1 cells were plated in a 96-well plate at a density of 10,000 cells per well and differentiated to macrophages with 20 ng/ml PMA. Following a 24-h exposure, the differentiation medium was replaced with fresh RPMI 1640, and cells were allowed to rest for 24 h. Then, 500 ng/ml of LPS was used to induce inflammation in these macrophages. The cells were treated for 24 h with different concentrations of DPK (1, 10, 100, 300, and 1,000 μg/ml) diluted in media. Cells were pretreated with DPK extract for 24 h before inducing inflammation with LPS. Cell supernatants were collected in a 96-well plate for measuring the levels of pro-inflammatory cytokines: TNF-α, IL-6, and IL-1β. Cytokine levels were measured using ELISA kits (BD Biosciences), according to the manufacturer’s protocol. Absorbance was recorded at 450 nm using an EnVision microplate reader (Perkin Elmer, United States). After washing the cells with PBS, 100 µl alamarBlue (15 μg/ml) was added to each well and incubated for 3 h at 37°C. Subsequently, fluorescence was measured using the EnVision microplate reader (Perkin Elmer, United States) at 530 (excitation) and 590 (emission) nm, and cell viability percentage was calculated.

### Experimental Animals

Six- to 8-week-old male CD-1 mice weighing 20–25 g were procured from Charles River Laboratory–licensed supplier, Hylasco Biotechnology Pvt. Ltd., Hyderabad, India. All animals were housed in polypropylene cages at a controlled temperature of 22 ± 3°C with a relative humidity of 60–70% under 12:12-h light and dark cycles in a registered animal house (registration number: 1964/PO/RC/S/17/CPCSEA). The animals were supplied with standard pellet diet (Purina Lab Diet, St. Louis, MO, United States) and sterile filtered water *ad libitum*. The study protocol was approved by the Institutional Animal Ethical Committee of Patanjali Research Institute vide IAEC approval number PRIAS/LAF/IAEC-069. All the experiments were performed as per the relevant guidelines and regulations.

### Divya-Peedantak-Kwath Sample Preparation and Dose Calculation for Animal Experiments

The animal equivalent doses of DPK for mice study was estimated based on the body surface area. The human recommended therapeutic dose of the DPK is 5 g powder boiled in 400 ml of water until ∼100 ml decoction remains, twice a day. Accordingly, total human dose is 10 g/70 kg/day. For DPK sample preparation, 500 g of DPK was boiled in 20 L distilled water and was reduced to 1.8 L of decoction, which was concentrated, filtered, and dried to yield 58.1 g of dry powder. Based on a decoction yield of 11.6%, the calculated human dose is 1.16 g/70 kg/day (16.6 mg/kg/day). Animal equivalent doses (mg/kg) for mouse were calculated by multiplying human equivalent dose (HED) (mg/kg) by a factor of 12.3 (Nair and Jacob, 2016). Resultant therapeutic equivalent doses for mouse were found to be 204 mg/kg. Therefore, 205 mg/kg (round off) has been taken as mouse mid-doses (HED). The low dose (69 mg/kg body weight) is one-third of the HED, and the high dose (615 mg/kg body weight) is three times the HED for mice.

### Chemotherapy-Induced Peripheral Neuropathy Animal Models and Treatment Groups

The CIPN animal model was generated as previously described (Thangamani et al., 2013; Kaur and Muthuraman, 2019). Eight- to 10-week old male CD-1 mice were randomized based on body weights and divided into six different groups consisting of six animals each. The details of randomization, dosage, and associated descriptions are depicted in Table 1. Due to one mortality observed in G3 (GABA), animal sample size was revised to *N* = 5. Animals of the group G1 served as normal control. PTX was administered intraperitoneally (i.p.) at 2 mg/kg body weight for six consecutive days to those of groups G2 to G6. PTX-treated animals were assessed for pain sensitivity. Animals that developed neuropathic pain were selected for the experiment and randomized into different treatment groups (G2–G6). Animals of group G2 served as disease control (DC) and were treated with 0.25% Na-CMC. The reference drug gabapentin (GABA) was administered intraperitoneally to the animals of group G3 (GABA) at 100 mg/kg body weight. Animals of groups G4 (DPK-69), G5 (DPK-205), and G6 (DPK-615) were treated orally with Divya-Peedantak-Kwath (DPK) at 69, 205, and 615 mg/kg body weight, respectively. All drug treatments were given for two weeks. The neurobehavioral monitoring of the animals through hot plate, tail flick latency, Von Frey, and Randall–Selitto tests was performed on different days, before and after PTX treatment. Body weights of the animals were measured every day for fifteen days (day 1–day 15) from the day when PTX administration was started. The change in body weight of each animal was calculated before and after the development of CIPN and after subsequent treatment. Animal feed and water consumption were recorded daily for the whole study duration.

**TABLE 1:** Group identification, dosage, and associated description used in *in vivo* study.

Group identity	Group name	Description	Treatment given (dosage)
G1	NC	Normal control	Normal saline (equivalent volume, p.o.)
G2	DC	Disease control: CIPN model established through intraperitoneal injection of paclitaxel (PTX)	PTX (2 mg/kg; i.p. qd×6)
G3	GABA	Treatment with gabapentin (GABA)	PTX (2 mg/kg; i.p. qd×6) + GABA (100 mg/kg; i.p.; qd×2W)
G4	DPK-69	Treatment with low dose (1/3 HED) of DPK	PTX (2 mg/kg; i.p. qd×6) + DPK (69 mg/kg; p.o.; qd×2W)
G5	DPK-205	Treatment with mid-dose (HED) of DPK	PTX (2 mg/kg; i.p. qd×6) + DPK (205 mg/kg; p.o.; qd×2W)
G6	DPK-615	Treatment with high dose (3× HED) of DPK	PTX (2 mg/kg; i.p. qd×6) + DPK (615 mg/kg; p.o.; qd×2W)

qd, quaque die (L) meaning “once a day”; p.o., per os (L) meaning “by mouth”; i.p., intraperitoneal; ×6, for 6 days; ×2W, for 2 weeks; HED, human equivalent dose.

### Assessment of Pain Behaviors

In order to establish the CIPN model, paclitaxel was administered intraperitoneally daily for 6 days. These 6 days are denoted as day 0–day 6. All the basal pain behaviors were assessed on day 0. Subsequently, hot plate assay was carried out on day 6, day 11, and day 16; tail flick and Von Frey assays on day 7, day 12, and day 17; and Randall–Selitto on day 9, day 13, and day 19.

#### Hot Plate Test

The hot plate test was performed to measure response latencies as described previously with minor modifications (Arrau et al., 2011; Balkrishna et al., 2019a). On the day of assessment, after 1 h of drug treatment, all the animals were placed in the Perspex cylinder of the hot plate (UgoBasile, Italy) maintained at 55.0 ± 0.5°C, and time to discomfort reaction (licking paws or jumping) was recorded as response latency. A cutoff point of 20°s was considered to avoid any possible accidental paw damage.

#### Tail Flick Test

The tail flick test as an acute model of pain was used to assess the antinociceptive effect of the drugs by measuring the latency of response. The tail flick test was executed with the modified method reported earlier using a plantar test device (7,370 plantar test; UgoBasile, Italy) at infrared radiation intensity of 50 with a cutoff time of 15 s (Keyhanfar et al., 2013; Balkrishna et al., 2019a). The animals were treated with different vehicle, standard, and test drug at different dose levels, and latency time was recorded at different days. The average of three readings with a gap of 5 min from each mouse was recorded in a blinded manner, by a researcher unaware of the treatment conditions.

#### Von Frey Test

The Von Frey test (Electronic Von Frey, UgoBasile, VA, Italy; model: 38,450-001) was used to assess responses to mechanical stimuli (Zanjani et al., 2014; Donvito et al., 2016). All the animals were habituated in transparent Perspex cubicles with elevated wire mesh bottom for 45 min for 3 consecutive days before starting the experiment. On the day of assessment, each mouse was acclimatized for 10 min before the test. The Von Frey filament (0.5 mm diameter) was applied to the plantar surface of the right hind paw of each mouse, with slowly increasing pressure. Responses like lifting, licking, or shaking of the paw was recorded as the paw withdrawal threshold (PWT; g) at different days. A PWT limit of 10 g was set to avoid tissue damage. Total three readouts with a gap of 3 min in between were recorded in a blinded manner, by a researcher unaware of the treatment conditions.

#### Randall–Selitto Pressure Test

The Randall–Selitto pressure test was performed to measure static hyperalgesia in animals according to the modified protocol described previously (Balkrishna et al., 2019b). The pain response as paw withdrawal threshold (PWT) to the mechanical stimulus was determined with a Randall and Selitto device on different days, after 1 h of drug treatment. PWT is defined as the force, applied to the dorsal surface of the left hind paw, that causes the mouse to vocalize or withdraw the paw. A limit of 25 g was set to avoid tissue damage. The average of three readings with a gap of 5 min in between was recorded in a blinded manner, by a researcher unaware of the treatment conditions.

### Histopathological Examination

Sciatic nerves were harvested from humanely sacrificed animals at the end of the experiment and stored in neutral buffered formalin for further processing for H&E staining according to procedure described earlier (Balkrishna et al., 2019a; Balkrishna et al., 2019b). The slides were examined under a microscope by a veterinary pathologist for histopathological lesions. The severity of the observed lesions was scored as 1 = minimal (<1%), 2 = mild (1–25%), 3 = moderate (26–50%), 4 = marked (51–75%), and 5 = severe (76–100%). The distribution of the lesions was recorded as focal, multifocal, and diffuse. Images of the histological slides (H&E) were captured at low (×10) and high (×40) magnifications using an Olympus Magnus microscope camera and processed by Olympus MagVision image analysis software.

### Antioxidant Profiling of Sciatic Nerve

The levels of reduced (GSH) and oxidized (GSSG) glutathione were measured according to Hissin and Hilf (1976). The isolated sciatic nerve tissue samples were homogenized in 0.1 M phosphate buffer (pH 8) containing 25% metaphosphoric acid. The homogenates were clarified through centrifugation at 10,000 g for 20 min at 4°C. Reduced glutathione (GSH) content was determined by incubating clarified homogenate with o-phthaldialdehyde (OPT) at room temperature for 15 min in dark. Fluorescence was measured at 350 (excitation) and 420 (emission) nm using the EnVision microplate reader (Perkin Elmer, United States). Levels of oxidized glutathione (GSSG) were determined by first incubating the clarified tissue homogenates with N-ethylmaleimide (0.04 M) for 30 min in dark and, subsequently, with 0.1 N NaOH and OPT solution for another 15 min. Fluorescence was measured at 350 (excitation) and 420 (emission) nm as mentioned earlier. Lipid peroxidation was monitored through the levels of malodialdehyde (MDA) in the clarified tissue homogenates according to Heath and Packer (1968). This assay is based on the reaction of MDA with thiobarbituric acid (TBA) forming an MDA–TBA adduct that absorbs at 532 nm. Clarified tissue homogenates were incubated with three volumes of 0.5% TBA (diluted in 0.1 M phosphate buffer, pH 8) at 95°C in a water bath for 25 min. The reaction was stopped by incubating the samples on ice. Absorbance was measured at 532 and 600 nm using the EnVision microplate reader (Perkin Elmer, United States). Absorbance values measured at 600 nm were subtracted from MDA–TBA complex values at 532 nm. MDA concentration was calculated using the Lambert–Beer equation with an excitation coefficient *ε* ^M^ = 155 mM^−1^cm^−1^.

### Assessment of Tumor Necrosis Factor-α Levels in the Serum

TNF-α levels in the serum were estimated using an ELISA kit (BioLegend, Inc., San Diego, CA, United States) according to the manufacturer’s instructions. The plate was read at 450 nm using the EnVision microplate reader (Perkin Elmer, United States).

### Statistical Analysis

Experiments were performed in triplicates and quantitative data represented as mean ± SEM. Two groups were compared by an unpaired *t*-test. For comparing three or more groups, one-way analysis of variance (ANOVA) followed by Dunnett’s post hoc test was used. For comparisons between three or more groups for different time points, two-way ANOVA followed by Tukey’s multiple comparisons test was used. Observations were considered statistically significant at *p*-value ≤ 0.05.

## Results

### Compositional Analysis of Divya-Peedantak-Kwath

**FIGURE 1:**
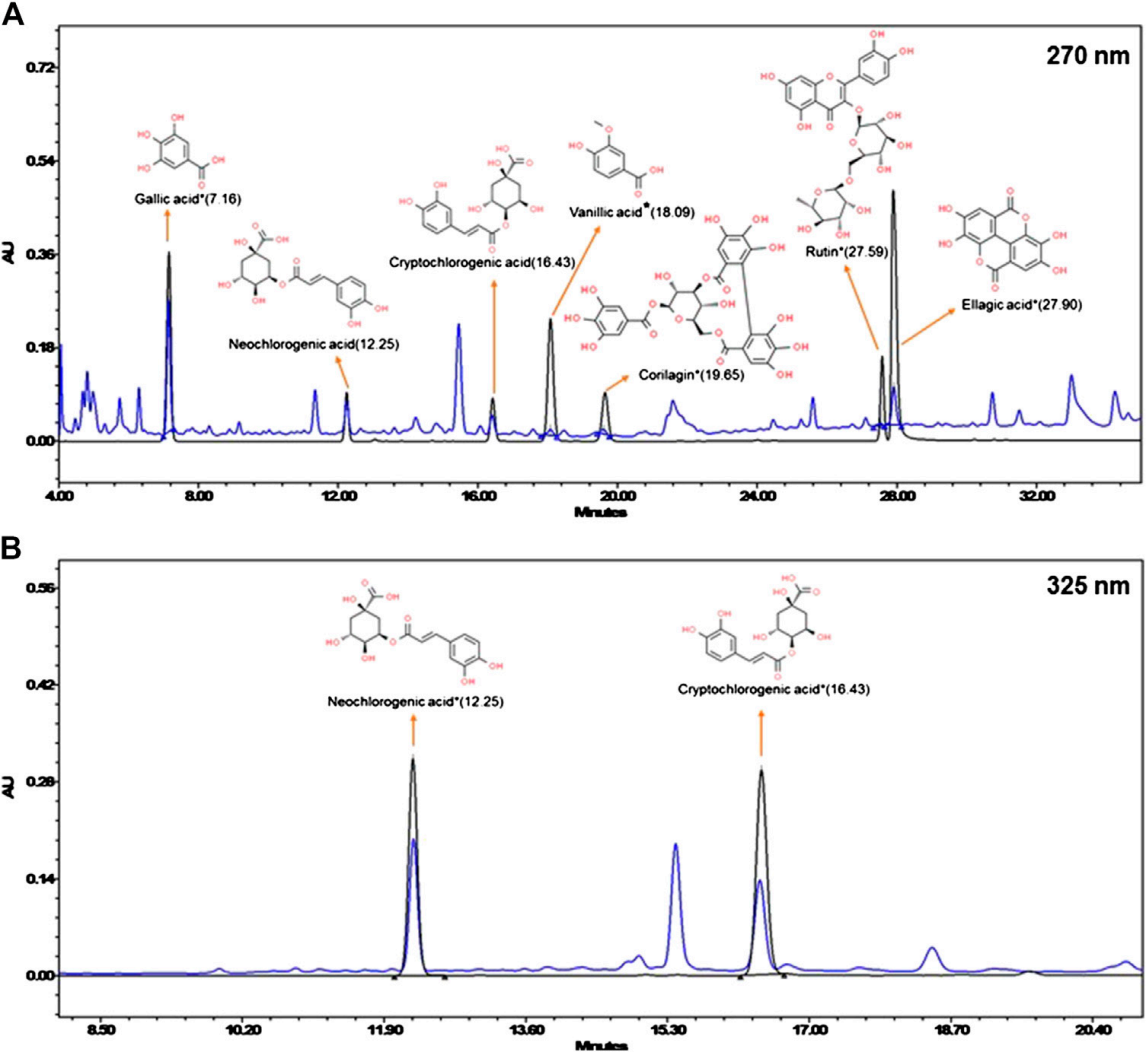
Chemical composition of Divya-Peedantak-Kwath (DPK). Overlap chromatographs of standard mix (in black) and Divya-Peedantak-Kwath (in blue) showing the peaks obtained pertaining to the active compounds present DPK. **(A)** shows the chromatograph for samples eluted at 270 nm, that is, gallic acid, vanillic acid, corilagin, rutin, and ellagic acid, whereas **(B)** shows those eluted at 325 nm, namely, neochlorogenic acid and cryptochlorogenic acid. Chemicals structures of the active compounds are provided along with the respective identified peaks (sourced from PubChem database). Quantification of these phytometabolites is shown in Table 2.

DPK is a herbal decoction consisting of 28 medicinally important plants (Table 2). Naturally, it is expected to be enriched with several active compounds. In fact, the herbs present in DPK are meticulously chosen to target the causative elements of CIPN, namely, oxidative stress, inflammation, and subsequent immune reactions. Therefore, it was imperative to check the active ingredients present in DPK that can target oxidative stress, reduce inflammation, and modulate immunological reactions, besides subduing the nociception and pain associated with CIPN. Employing the high-performance liquid chromatographic (HPLC) technique, the major compounds identified in DPK are gallic acid (1.009 μg/mg) and neochlorogenic acid (1.067 μg/mg). Besides, cryptochlorogenic acid (0.804 μg/mg), ellagic acid (0.243 μg/mg), corilagin (0.124 μg/mg), vanillic acid (0.078 μg/mg), and rutin (0.069 μg/mg) were also detected (Figures 1A,B; Table 3).

**TABLE 2:** Herbal components of Divya-Peedantak-Kwath (DPK).

S.No	Botanical name	Common name	Part used	Quantity in DPK (g/100 g)
1	*Nyctanthes arbor-tristis* L.	Parijat	Leaf	19.5
2	*Vitex negundo* L.	Nirgundi	Leaf	19.3
3	*Pluchea lanceolate* (DC.) C. B. Clarke	Rasna	Leaf	19.5
4	*Gossypium herbaceum* L.	Binaula	Seed	3.9
5	*Withania somnifera* (L.) Dunal	Ashwagandha	Root	3.9
6	*Cyperus scariosuso* R.Br.	Nagarmotha	Root	3.9
7	*Sida cordifolia* L.	Bala panchang	Whole plant	3.9
8	*Boerhavia diffusa* L.	Punarnava	Whole plant	3.9
9	*Trachyspermum ammi* (L.) Sprague	Ajwain	Seed	3.9
10	*Zingiber officinale* Roscoe	Sonth	Rhizome	0.8
11	*Ricinus communis* L.	Erand	Root	0.8
12	*Cedrus deodara* (Roxb. Ex D. Don) G. Don	Devdaru	Stem	0.8
13	*Curcuma zedoaria* (Christm.) Roscoe	Kachur	Rhizome	0.8
14	*Acorus calamus* L.	Gudbach	Rhizome	0.8
15	*Tribulus terrestris* L.	Gokshur	Fruit	0.8
16	*Aconitum palmatum* D. Don	Atis meetha	Root	0.8
17	*Cassia fistula* L.	Amaltas	Fruit	0.8
18	*Asparagus racemosus* Willd.	Satavar	Root	0.8
19	*Piper longum* L.	Pippli chhoti	Fruit	0.8
20	*Holarrhena antidysenterica* (Roth) Wall. Ex A. DC.	Kuda chaal	Bark	3.9
21	*Coriandrum sativum* L.	Dhaniya	Fruit	0.8
22	*Terminalia chebula* Retz.	Harad chilka	Fruit	0.8
23	*Piper retrofractum* Vahl	Chavya	Root	0.8
24	*Tinospora cordifolia* (Willd.) Miers	Giloy	Stem	0.8
25	*Argyreia speciosa* (L. f.) Sweet	Vidhara	Stem	0.8
26	*Foeniculum vulgare* Mill.	Sounf	Fruit	0.8
27	*Solanum indicum* var. *lividum* (Link) Bitter	Kateli badi	Whole plant	0.8
28	*Solanum surattense* Burm. f.	Kateli chhoti	Whole plant	0.8

**TABLE 3:** Active phytometabolites present in Divya-Peedantak-Kwath (DPK), as per the HPLC chromatographs shown in Figure 1.

S.No	Name of marker compound	Quantity in DPK (µg/mg)
1	Gallic acid	1.009
2	Neochlorogenic acid	1.067
3	Crypto chlorogenic acid	0.804
4	Vanillic acid	0.078
5	Corilagin	0.124
6	Rutin	0.069
7	Ellagic acid	0.243

### Divya-Peedantak-Kwath Blocks Lipopolysaccharide-Induced Inflammation in Differentiated Macrophages

**FIGURE 2:**
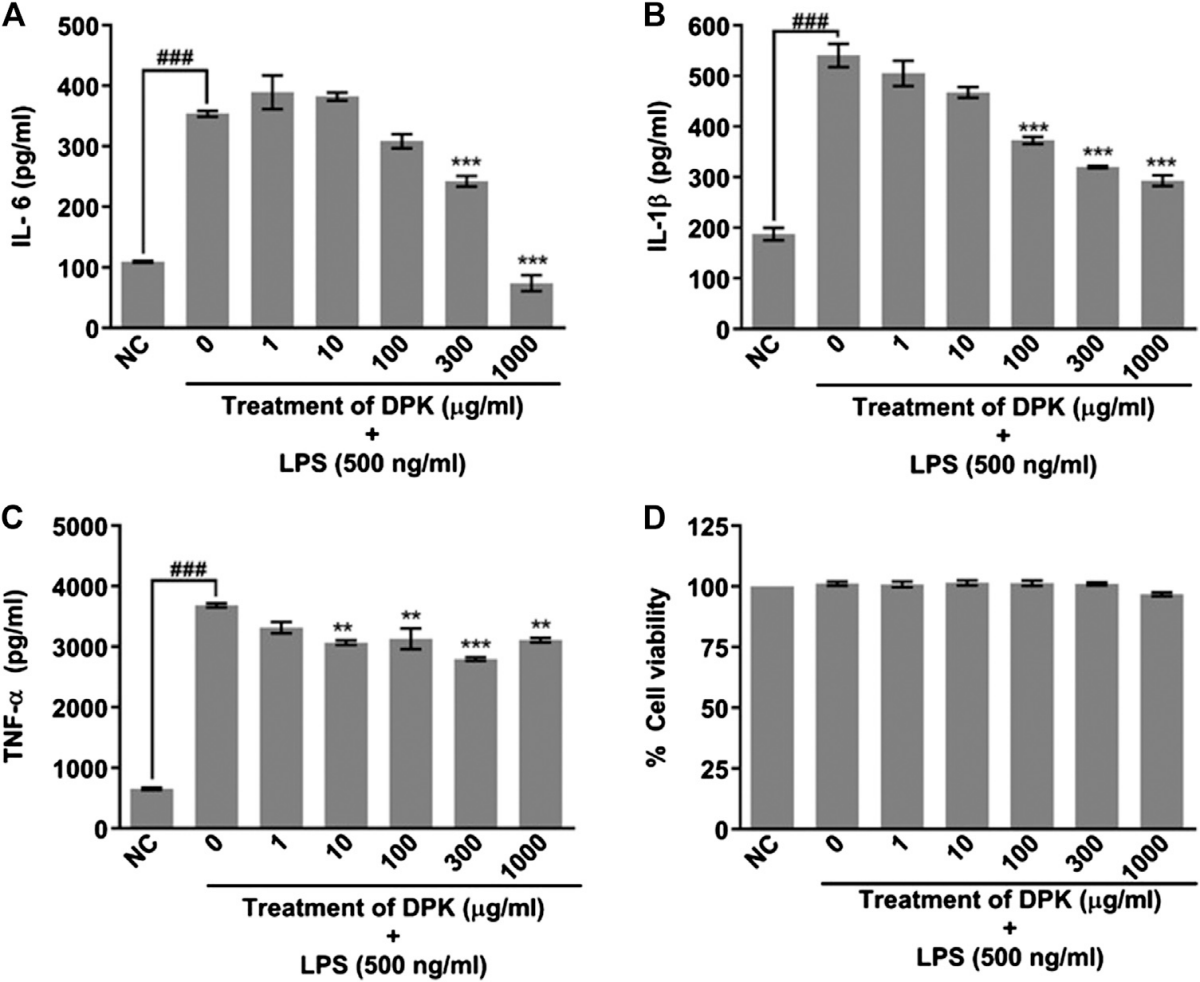
DPK has anti-inflammatory effects with acceptable cytosafety. Anti-inflammatory activity of DPK was evaluated through its effect on the elevated levels of pro-inflammatory cytokines, like IL-6 **(A)**, IL-1β **(B)**, and TNF-α **(C),** in case of LPS (500 ng/ml)-induced inflammation in PMA-differentiated THP-1 cells. Absence of cytotoxicity and suitability of the established system for evaluating anti-inflammatory activity was confirmed through alamarblue assay **(D)**. Differentiated THP-1 cells without any LPS treatment was taken as normal control (NC). LPS-treated cells that did not receive DPK treatment (0) served as disease control for comparing the observed anti-inflammatory effect of DPK. Cytokine levels in this group were compared with NC to confirm the credibility of the *in vitro* inflammation model. Data are represented as mean ± SEM of three independent experiments. Statistical analysis was done using one-way ANOVA followed by Dunnett’s multiple comparisons test and observation represented as ^###^ *p* < 0.001, when significantly different in comparison to NC; **p < 0.01, ***p < 0.001, when significantly different from DPK-untreated (0) group induced with inflammation.

Given the fact that DPK is practically a storehouse of anti-inflammatory agents, we evaluated its efficiency in attenuating inflammation on differentiated THP-1 cells *in vitro.* PMA-differentiated, LPS-inflamed human THP-1 cells were treated with different concentrations (1, 10, 100, 300, and 1,000 μg/mg) of DPK. LPS-treated and -untreated differentiated THP-1 cells, respectively, serving as disease (0) and normal (NC) controls did not receive any DPK treatment. Pro-inflammatory cytokines, namely, interleukin-6 (IL-6), interleukin 1 beta (IL-1β), and tumor necrosis factor-alpha (TNF-α) have been implicated in neuroinflammation and neuropathic pain (Leung and Cahill, 2010; del Rey et al., 2012; Hung et al., 2017). Therefore, we monitored the effect of DPK treatment on these cytokines released in LPS-induced THP-1 cells. The levels of IL-6, IL-1β, and TNF-α were, respectively, 3, 2.8, and 5.6 times higher in disease control (0) cells with inflammation (Figures 2A–C). Noticeable reductions in IL-6 levels from 353.9 (±4.7) pg/ml in (0) group to 242.2 (±8.8) pg/ml and 74.1 (±13.1) pg/ml were observed when the cells were treated with DPK at 300 and 1,000 μg/mg concentrations, respectively (Figure 2A). Effects on IL-1β levels were visible with DPK treatments at 100 μg/mg or more. It reduced from 540.83 (±22.8) pg/ml in (0) group to 344 (±28.4), 320 (±2.3), and 293 (±27.6) pg/ml, when treated with 100, 300, and 1,000 μg/ml of DPK, respectively (Figure 2B). Significant reduction in the level of TNF-α relative to the disease control (0) cells (3,685.0 ± 33.3 pg/ml) was observed with 10 μg/mg DPK treatment (3,070.1 ± 36.9 pg/ml) onward. However, we did not observe dose dependency in the reduction in TNF-α (Figure 2C). Taken together, these observations showed that DPK possess strong anti-inflammatory effects. In order to ensure that PMA, LPS, DPK, or their cotreatments did not have any untoward effects on the cells and that the model was suitable for evaluation of inflammation, cell viability of THP-1 cells was checked through alamarBlue assay, conforming to the described experimental regimen. Cells were equally viable in the control and experimental groups, confirming the cytosafety of DPK in the treatment regimen and also lending the credibility to the experimental setup (Figure 2D).

### Divya-Peedantak-Kwath Treatment Prevented Paclitaxel-Induced Weight Gain without Affecting Regular Metabolism

**FIGURE 3:**
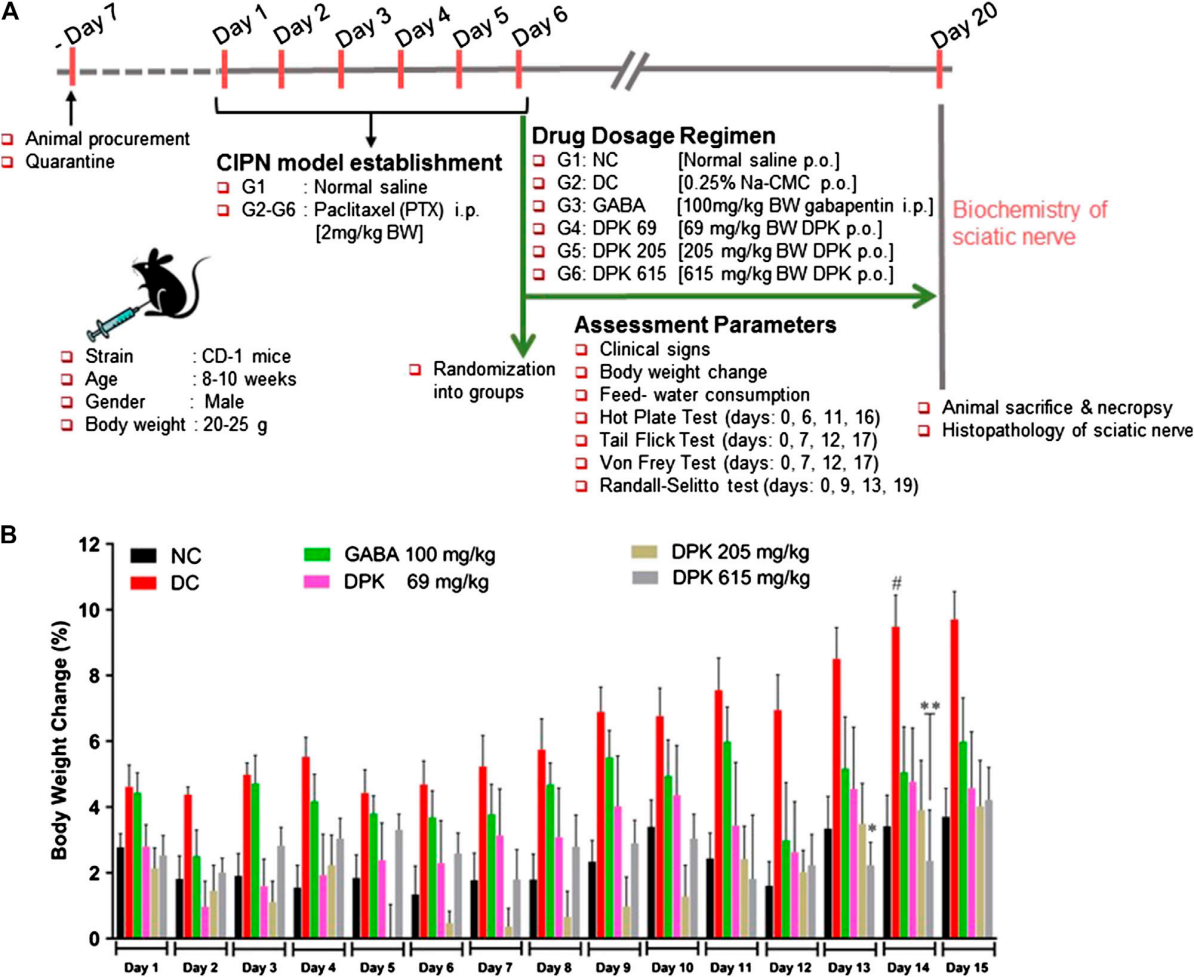
*In vivo* experimental design and mouse CIPN model establishment. **(A)** Schematic representation summarizing details of animal procurement, quarantining, CIPN model establishment through i.p. injections of PTX (2 mg/kg body weight/day for 6 days), drug administration, assessment parameters, and *ex vivo* experiments. The groups included in this study are normal control (NC), disease control (DC) (PTX-treated animals receiving no treatment), gabapentin (GABA) (PTX-treated animals receiving reference drug gabapentin intraperitoneally at 100 mg/kg body weight/day for 2 weeks), DPK 69 (PTX-treated animals receiving 69 mg/kg body weight of DPK orally per day for 2 weeks), DPK 205 (PTX-treated animals receiving 205 mg/kg body weight of DPK orally per day for 2 weeks), and DPK 615 (PTX-treated animals receiving 615 mg/kg body weight of DPK orally per day for 2 weeks). **(B)** Body weights of the animals in different groups receiving different treatments were monitored for 15 days and graphically represented. Data are represented as mean ± SEM, where *N* = 6 in all groups, except GABA, where *N* = 5. Data were statistically analyzed using two-way ANOVA followed by Tukey’s multiple comparisons test and observation represented as ^#^ *p* < 0.05, when significantly different in comparison to NC; *p < 0.05, **p < 0.0, when significantly different in comparison to DC.

The mouse model of CIPN was generated through intraperitoneal injection of PTX at a human-relevant clinical dose. The experimental design and regimen have been explained in detail in the *Material and Methods* (*Chemotherapy-Induced Peripheral Neuropathy Animal Models and Treatment Groups*), Table 1, and schematically represented in Figure 3A. The effect of DPK on general health and metabolism was monitored through change in the body weights of the animals and alteration in their food and water intake habits. The PTX-induced disease control (DC) group showed approximately 9% increase in body weight by day 14, whereas that of normal control (NC) group was 3% by the same day. The DC group showed a statistically significant more increase in body weight than the normal control (NC) animals. Interestingly, an increase in body weights was under control by day 13 and day 14 in the DPK-615 group, receiving a daily oral dose of 615 mg/kg DPK. The average body weights in this group were significantly lower when than those of the DC and NC groups. However, similar effects were not observed in DPK-69 and DPK-205 groups, which received daily oral doses of 69 and 205 mg/kg of DPK, respectively. This effect was also not observed in the group receiving gabapentin, the FDA-approved drug for treating CIPN (Figure 3B). Food and water intake were similar among the animals across different groups (data not shown). This suggested that observed increase in the body weight of the DC group is not due to any change in the eating and drinking habits of the animals, rather most likely due to some underlying metabolic alterations experienced while establishing the CIPN mouse model.

### Divya-Peedantak-Kwath Treatment Modulated Pain Responses

**FIGURE 4:**
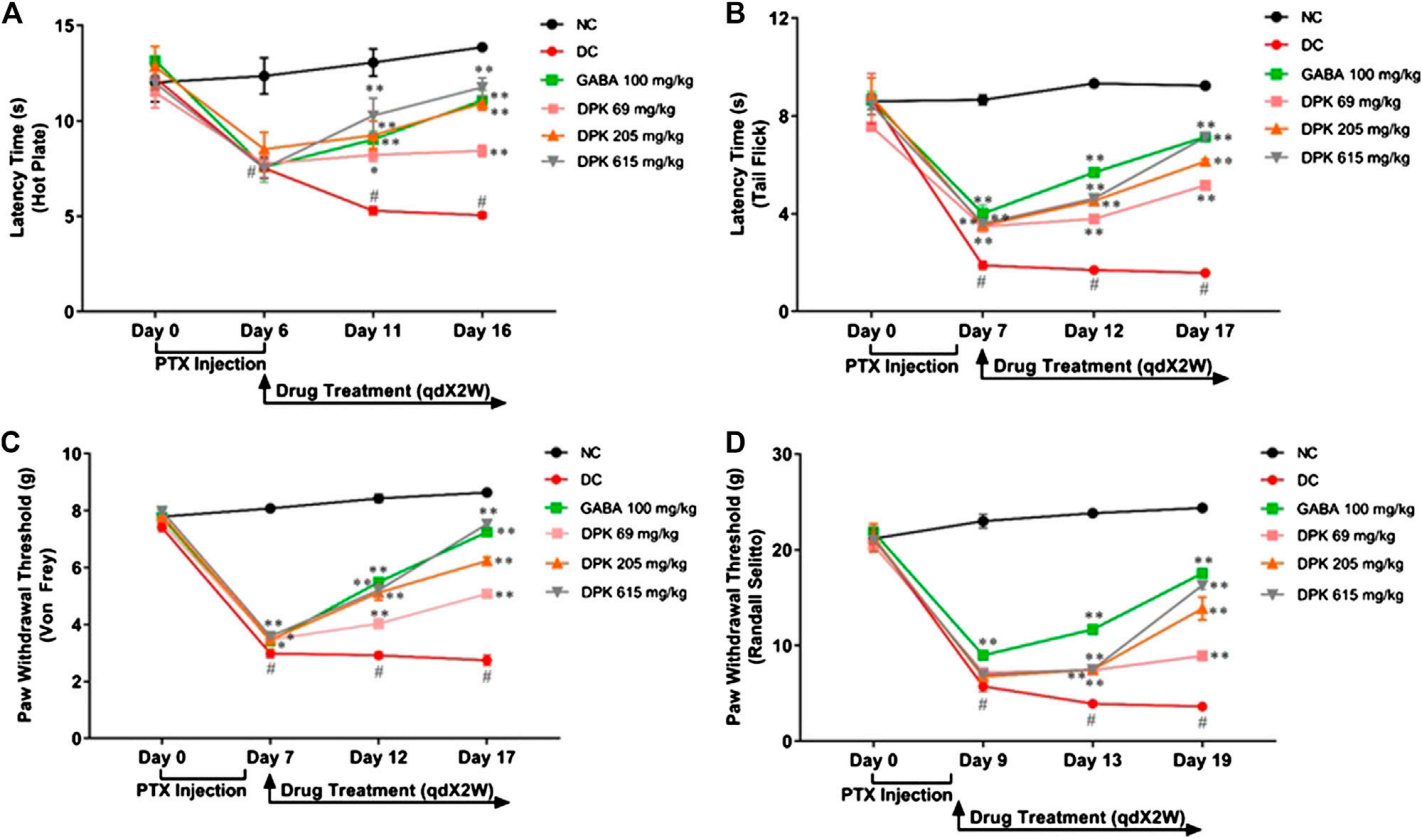
Alterations in nociceptive behavior in response to DPK treatment. Modulations of various pain response behaviors by DPK treatments were evaluated by using the hot plate **(A)**, tail flick latency **(B)**, Von Frey **(C)**, and Randall–Selitto **(D)** tests and observation represented through individual graphs for each test. qdX2W is to be read as on prescription recommended dose of X drug (in this case DPK or gabapentin) for 2 weeks (2W). Data are represented as mean ± SEM, where *N* = 6 in all groups, except GABA, where *N* = 5. Data were statistically analyzed using two-way ANOVA followed by Tukey’s multiple comparisons test and observation represented as ^#^ *p* < 0.05, when significantly different in comparison to NC; *p < 0.05, **p < 0.01, when significantly different in comparison to DC.

PTX has been shown to induce heat hyperalgesia, mechano-hyperalgesia, mechano-allodynia, and allodynia (Scripture et al., 2006). Therefore, the pharmacological effects of DPK on PTX-induced neuropathic pain sensitivity was analyzed using the hot plate test, tail prick latency test, Von Frey test, and Randall–Selitto test (Figures 4A–D). Efficacy of DPK in assuaging heat hyperalgesia was monitored through the hot plate test (Figure 4A). PTX administration reduced the latency times to almost half (from average 12.29 ± 0.24 s to around 7.78 ± 0.18 s) by day 6 in all the groups, except NC, one that did not receive the chemotherapeutic treatment. A significant increase in the latency times of the groups receiving treatments for CIPN was observed over time. Latency time of the group receiving gabapentin increased from an average 7.58 ± 0.80 to 9.03 ± 0.45 s by day 11, a significant improvement over the DC group, in which the latency time further reduced to around 5.29 (±0.20) s over this period. An increase in latency time (8.52 ± 0.88 to 9.24 ± 0.75 s) in the group receiving DPK at 206 mg/kg body weight was comparable to the GABA group at day 11. However, the group receiving DPK at 69 mg/kg body weight showed only a marginal increase (7.76 ± 0.39 to 8.22 ± 0.33 s) in latency time over this period. The most significant increase in latency time, from 7.52 (±0.88) to 10.28 (±0.91) s, was observed in the group that received DPK at 615 mg/kg body weight. This increase was even more pronounced than that observed in the GABA group. Moreover, the increase in latency times of all the treatment groups was significant when compared to the DC group. The improvement was more noticeable by day 16 which witnessed a sharp increase in latency times 11.76 (±0.49) and 10.92 (±0.36) s, respectively, for DPK-615 and DPK-205 groups. The GABA group showed a trend similar to the DPK-205 group, with its average latency time increasing to 11.08 (±0.53) s. However, DPK-69, with its average latency time increasing to only 8.43 (±0.3) s, did not show a sharp increase in latency time as other DPK groups, although it was statistically significant (*p* < 0.01) relative to the DC group. The increase in latency times observed in all other treatment groups was significantly more when compared to DC.

The tail flick latency test was conducted to evaluate the modulation of nociception and relieving of mechano-hyperalgesia by DPK treatment (Figure 4B). A threshold around 8 (±0.18) s of latency was established which was maintained in the NC group. Induction of CIPN with PTX reduced this threshold significantly, to an average 3.65 (±0.12) s in the treatment groups by day 7. In the DC group, this was even lower (1.89 ± 0.17 s). This reduction of latency threshold upon CIPN induction reflects allodynia. Treatment with gabapentin (GABA group) increased the average latency time to 5.69 ± 0.09 s by day 12 and 7.15 ± 0.09 s by day 17. Latency time in the DPK-615 group was around 3.6 ± 0.11 s by day 12 but caught up with the GABA group at 7.13 ± 0.08 s by day 17. These values were noticeably higher than those of the DC group. The increase in latency times in DPK-69 and DPK-205 trailed behind. DPK-205 witnessed an increase in latency time to 4.53 ± 0.15 s by day 12 and 6.14 ± 0.09 s by day 17. Latency times for DPK-60 after day 12 and day 17 were around 3.79 (±0.17) and 5.16 (±0.13) s, respectively. While all the treatment groups showed increases in latency times reflecting amelioration of the inflicted allodynia, latency time of the DC group remained significantly lower than that of the treatment groups. In fact, it reduced to 1.70 (±0.05) s by day 12 from 1.89 (±0.17) s on day 7 and further to 1.58 (±0.08) s by day 17, although the reduction was not visibly discernible.

The effect of DPK treatment in ameliorating mechano-allodynia due to PTX-induced peripheral neuropathy was evaluated by using the Von Frey test (Figure 4C). The average paw withdrawal threshold (PWT) for all the groups was around 7.72 ± 0.08 g before PTX treatment. PWT reduced to average 3.49 ± 0.03 g by day 7 in the treatment groups once the PTX dosing was initiated. For this DC group, this was even lower at 2.99 ± 0.15 g. These were significant reductions across the experimental groups when compared to the PWT of the NC group that continued to be around 8.43 ± 0.15 g till day 12, showing a marginal increase by day 17 (8.64 ± 0.09 g). PWT increased in the GABA group to 5.49 ± 0.12 g by day 12 and 7.25 ± 0.04 g by day 17. The increase in PWT in the DPK-615 group also followed a trend similar to that of the GABA group (5.21 ± 0.26 g by day 12 and 7.53 ± 0.16 g by day 17). The PWT increase in DPK-205 was similar to that of GABA and DPK-615 groups till day 12 (5.12 ± 0.26 g), beyond which it fell behind to 6.24 ± 0.13 g by day 17. A discernible increase in PWT in the DPK-69 group was observed only by day 17 (5.08 ± 0.14 by day 17 vs. 3.47 ± 0.05 g of day 7). However, PWTs of all these groups were significantly more than those of the DC group, in which the threshold kept decreasing, albeit, marginally.

Allodynia or increased pain sensation in PTX-induced neuropathy can be due to neuroinflammation (Myers et al., 2006; Ji et al., 2018). Therefore, the Randall–Selitto test was used to assess the effect of DPK treatment on modulation of this pain sensitivity (Figure 4D). While the normal PWT in all the groups was observed to be 21.2 ± 0.18 g, it reduced to around 5.5–9 g after PTX dosing by day 9. Following gabapentin treatment, PWT increased to 11.69 ± 0.51 and 17.54 ± 0.31 g by day 13 and day 19, respectively. A visible increase in PWT in case of DPK-69, DPK-205, and DPK-615 groups was observed to be 8.93 ± 0.22, 13.86 ± 1.17, and 16.31 ± 0.45 g, respectively, only by day 19. Meanwhile, PWT in the DC group continued to decrease further to 3.93 ± 0.23 and 3.63 ± 0.22 g by day 13 and day 19, respectively. All of these observations demonstrated that DPK can be a very potent modulator of PTX-induced pain sensitivity.

### Divya-Peedantak-Kwath Treatment Attenuated Oxidative Stress and Inflammation *In Vivo*

**FIGURE 5:**
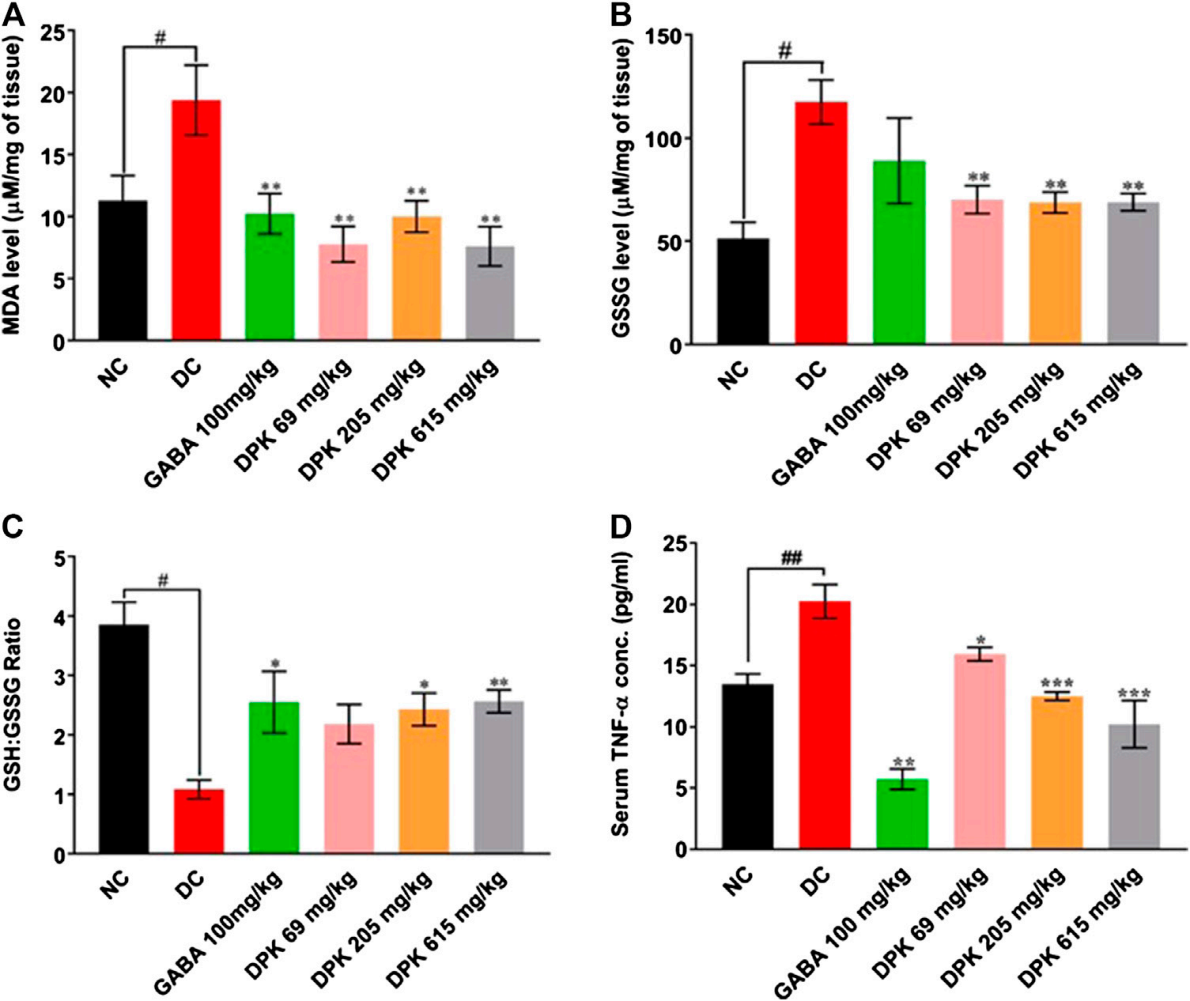
DPK alleviates CIPN-induced oxidative stress and inflammation *in vivo*. Effect of DPK treatments on the oxidative stress status of the nerve tissue was evaluated through MDA level **(A)**, GSSG level **(B)**, and GSH:GSSG ratio **(C)**. Observations are represented through individual graphs for each parameter. **(D)** Anti-inflammatory effect of DPK treatment was evaluated through serum levels of TNF-α. Data are represented as mean ± SEM, where *N* = 6 in all groups, except GABA, where *N* = 5. Data were statistically analyzed using one-way ANOVA followed by Dunnett’s multiple comparisons test and observation represented as ^#^ *p* < 0.05, when significantly different in comparison to NC; *p < 0.05, **p < 0.01, when significantly different in comparison to DC.

The effect of DPK treatment on PTX-induced oxidative stress in peripheral nerve tissue was evaluated *ex vivo* by monitoring tissue levels of MDA, GSSG, and GSH:GSSG (Figures 5A–C). The DC group is expected to experience enhanced lipid peroxidation due to PTX treatment which was reflected as a significantly increased MDA level (*p* < 0.01) when compared to the NC group. Gabapentin and DPK treatments significantly (*p* < 0.01) reduced the MDA levels in the respective treatment groups (Figure 5A). As expected, the GSSG level in the DC group was more than double of that observed in the NC group (117.34 ± 10.61 vs. 51.45 ± 7.71 μM/mg of tissue, *p* < 0.05). Treatment with gabapentin although showed visible reduction in the GSSG level, the change was not statistically significant when compared to that of the DC group. However, GSSG levels in all the DPK-treated groups were significantly lower (*p* < 0.01) relative to DC groups (Figure 5B). Likewise, the GSH:GSSG in all the treatment groups were noticeably high when compared to the DC group. The differences were statistically significant (*p* < 0.05 for GABA and DPK-205 groups and *p* < 0.01 for the DPK-615 group). GSH:GSSG for the DPK-69 group was visibly more than that of the DC group, but the difference was not statistically significant (Figure 5C). We also checked the effect of DPK treatment on the serum levels of pro-inflammatory cytokine, TNF-α in study animals (Figure 5D). The mean serum TNF-α was significantly raised in the DC group when compared to the NC group (20.25 ± 1.36 pg/ml vs. 13.50 ± 0.83 pg/ml, *p* < 0.01). Treatment with gabapentin reduced the serum TNF-α level to 5.72 ± 0.84 pg/ml (DC vs. GABA, *p* < 0.001). Treatments with DPK showed a dose-dependent reduction of serum TNF-α, and the changes were all statistically significant when compared to the DC group [20.25 ± 1.36 pg/ml in DC vs. 15.94 ± 1.36 (*p* < 0.05), 12.50 ± 0.34 (*p* < 0.001), and 10.23 ± 1.93 (*p* < 0.001) pg/ml in DPK-69, DPK-205, and DPK-615 groups, respectively]. Taken together, these observations indicate that DPK treatment alleviates the oxidative stress and concomitant inflammation imposed by PTX treatment.

### Divya-Peedantak-Kwath Assuages Etiology of Paclitaxel-Induced Neuropathy in Sciatic Nerve

**FIGURE 6:**
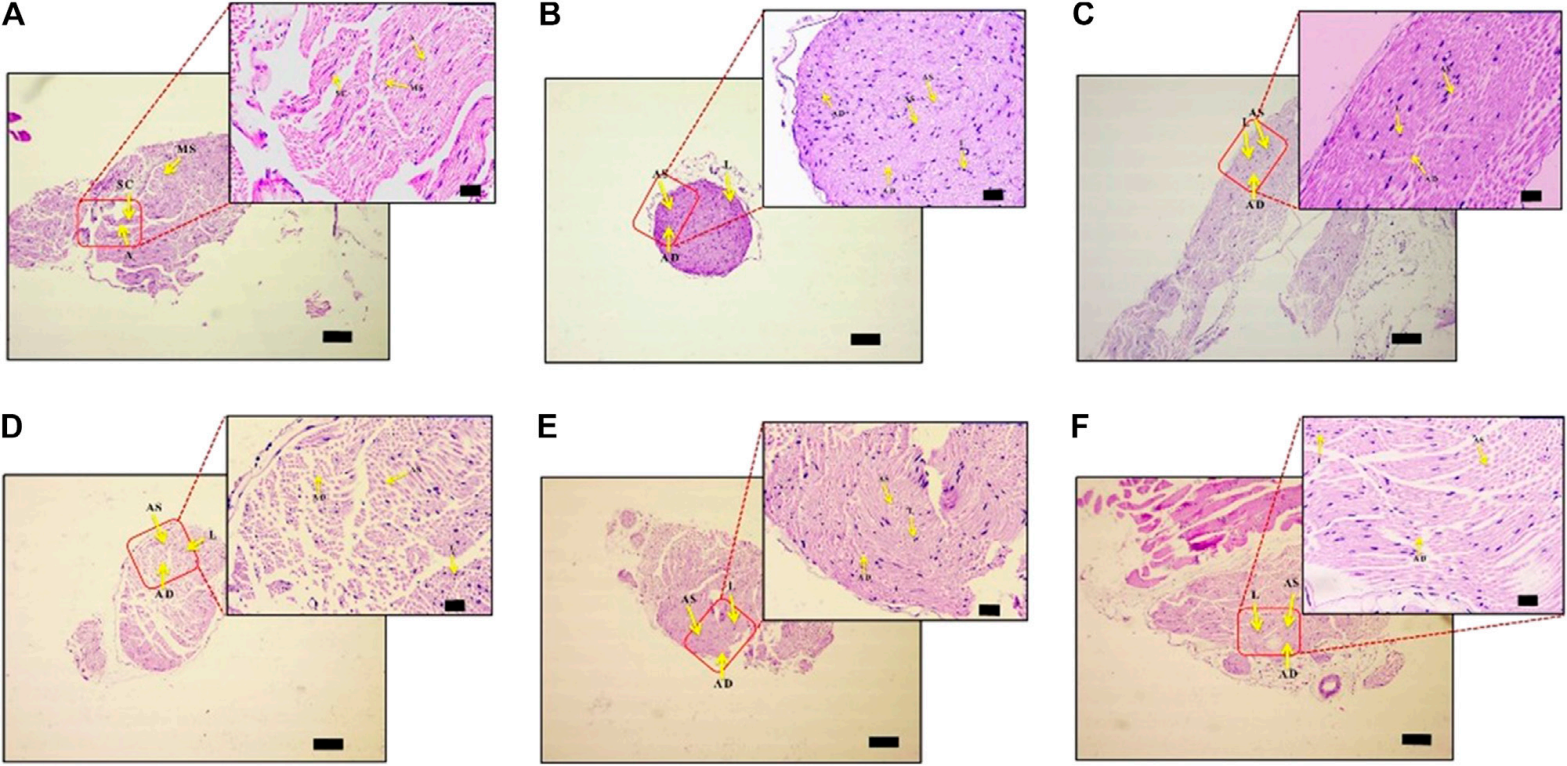
DPK treatment protects nerve tissues from histopathology of neuropathic pain. Effect of DPK treatments on CIPN histopathology was evaluated through H&E staining for axonal degeneration (AD), swelling (AS), and lymphocytic infiltration (L). Representative micrographs of H&E stained sciatic nerve sections from NC **(A)**, DC **(B)**, GABA **(C)**, DPK 69 **(D)**, DPK 205 **(E)**, and DPK 615 **(F)** groups at low (×10; scale bar: 100 µm) and high (×40; scale bar: 20 µm) magnifications are provided. Intact axons are indicated as “A,” Schwann cells as “SC,” myelin sheath as “MS,” axonal degeneration as “AD,” axonal swelling as “AS,” and lymphocytic infiltration as “L” in these micrographs.

**FIGURE 7:**
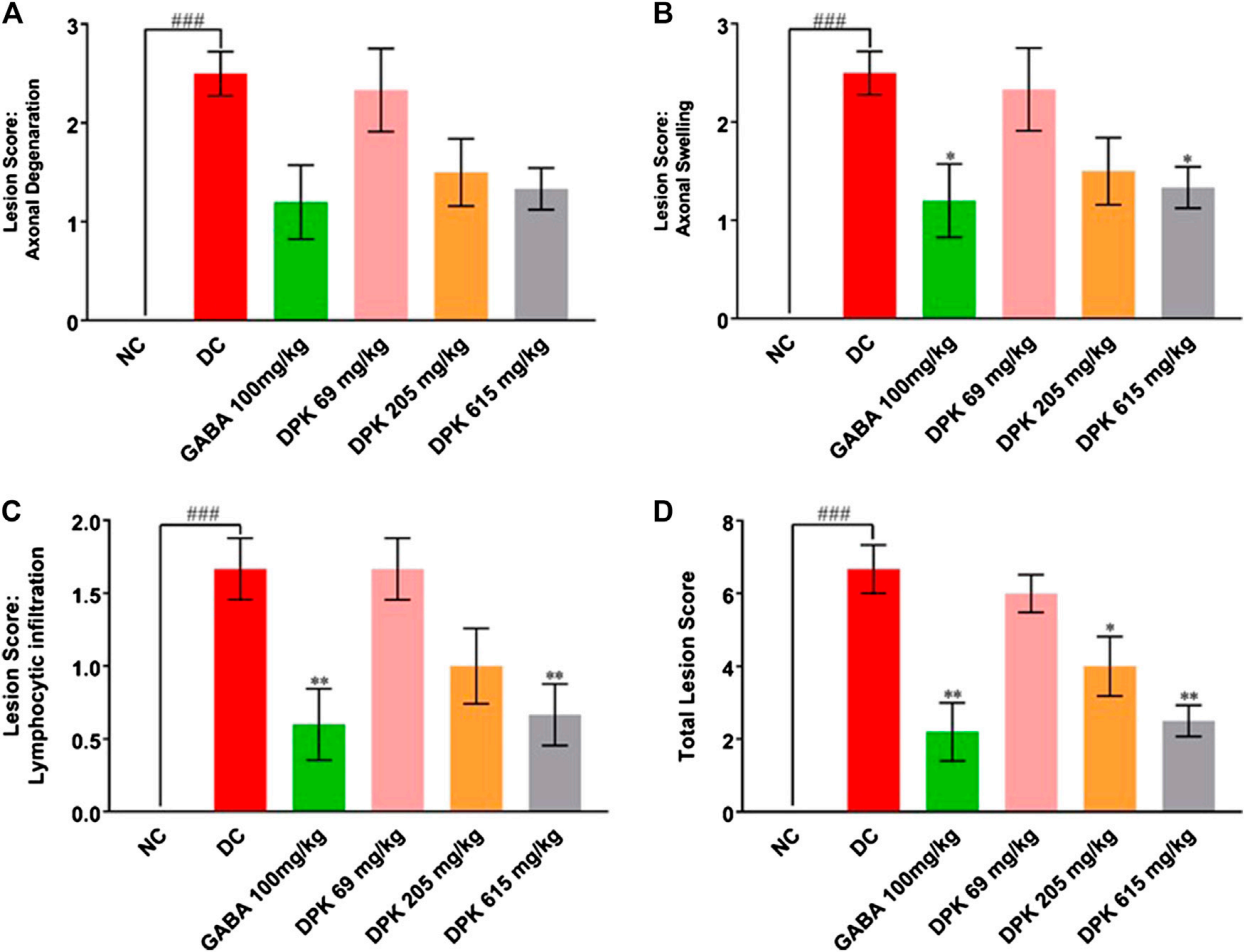
DPK treatment ameliorates CIPN etiology. Effectivity of DPK treatment on reducing CIPN etiology is quantitatively represented as lesion scorings for axonal degeneration **(A)**, axonal swelling **(B)**, and lymphocytic infiltration **(C)**. Lesion scores are represented through individual graphs for respective parameters and total score **(D)**. Data represented as mean ± SEM, where *N* = 6 in all groups, except GABA, where *N* = 5. Data were statistically analyzed using one-way ANOVA followed by Dunnett’s multiple comparison test and observation represented as ^###^ *p* < 0.001, when significantly different in comparison to NC; *p < 0.05, **p < 0.01, when significantly different in comparison to DC.

PTX causes axonal degeneration, swelling, and lymphocytic infiltration (Peters et al., 2007; Tasnim et al., 2016). Thus, efficacy of DPK in treating these conditions was evaluated through histological staining of sciatic nerve tissue and subsequent microscopic analyses (Figure 6). These pathological features have been scored and quantitatively represented in Figures 7A–D. Representative micrograph from the NC group shows intact axons (A), Schwann cells (SC), and myelin sheath (MS) (Figure 6A). Consequently, no lesion score showed up for this group (Figures 7A–D). PTX treatment shows degenerated and swollen axons, indicated as AD and AS, respectively, in the micrograph. The image also shows infiltrated lymphocytes (L) (Figure 6B). Treatments with gabapentin and DPK (at 205 and 619 mg/kg body weight doses) showed visible reduction in these pathological features in the respective images (Figures 6C,E,F). However, the group receiving DPK at 69 mg/kg body weight did not show much improvement with respect to these features (Figure 6D). Individual lesion scores for axonal degeneration, swelling, and lymphocytic infiltration as well as the total one for the DC group were obviously the maximum. The lesion score for axonal degeneration although reduced visibly in GABA group, the change was not statistically significant. Similarly, reductions in the lesion score of axonal degeneration observed in DPK-treated groups were also not statistically significant. The changes in DPK-205 and DPK-619 groups were comparable to those in the GABA group (Figure 7A). Lesion scores for axonal swelling in both GABA and DPK-619 groups were significantly lower (*p* < 0.05) when compared to the DC group. Although, the lesion score for axonal swelling in the DPK-205 group was comparable to GABA and DPK-619 groups, it did not show statistical significance when compared to the DC group. Lesion score for axonal swelling in the DPK-69 group did not show much reduction and was comparable to the DC group (Figure 7B). Lesion scores for infiltrating lymphocytes for each group exhibited a pattern similar to that shown by the lesion score for axonal swelling, except that the reduction in infiltrating lymphocytes in case of GABA and DPK-619 groups were more pronounced (*p* < 0.01) when compared to the DC group (Figure 7C). In the total lesion scoring, in addition to the GABA and DPK-619 groups, the DPK-205 group also showed statistically significant reduction relative to the DC group. However, the reduction in the latter was less pronounced than the former ones (*p* < 0.05 in case of DPK-205 vs. *p* < 0.01 in cases of GABA and DPK-619). Total lesion score for DPK-69 did not show statistically significant reduction when compared to the DC group, rather total lesion scores for both appeared comparable (Figure 7D). Altogether these observations showed that DPK treatment, albeit at higher doses, can assuage the etiology of PTX-induced neuropathy. Considering that DPK is an herbal decoction, higher dose may not be an issue with respect to toxicity. In fact, the recommended doses of gabapentin have reported side effects which may be not the case for DPK.

## Discussion

A thorough understanding of the disease pathophysiology is mandatory in order to identify a better treatment for CIPN. Agents benefitting diabetic peripheral neuropathic pain are often ineffective against CIPN, implicating different disease mechanisms in case of these two peripheral neuropathies (Frampton and Foster, 2006; Finnerup et al., 2010; Mishra et al., 2012). However, out of pregabalin and gabapentin, approved by the Food and Drug Administration (FDA) for the first-line treatment of diabetic neuropathic pain, gabapentin has been found to be effective against PTX-induced peripheral neuropathy in a recent randomized clinical trial (Aghili et al., 2018) Both these drugs are well tolerated, although somnolence and dizziness are most common side effects. Peripheral edema, weight gain, nausea, vertigo, asthenia, dry mouth, and ataxia are some other side effects. Nevertheless, there are reports on these side effects resolving with time or dose reduction (Fallon and Colvin, 2013). The gabapentin-treated group in our study did not show any weight gain.

About 20% of cancer pains are treatment related and are mostly neuropathic in nature (Verstappen et al., 2003). Neurotoxic effects of chemotherapy lead to CIPN. This distributes pain in a stocking-and-glove manner (Luo et al., 2001). The pain is pricking or burning in nature with “electric sensation” (DeSantis et al., 2015). At molecular levels, dynamics of microtubule assembly is affected in PTX-induced CIPN (Chaudhry et al., 2008). Taxane agents bind to polymerized tubulin within microtubules and prevent their depolymerization (Jordan and Wilson, 2004). This leads to microtubule aggregation in axons and Schwann cells (Kuroi and Shimozuma, 2004; Stillman and Cata, 2006; Izycki et al., 2016; Ghoreishi et al., 2018). Axonal degeneration is a common feature in CIPN pathology (Fukuda et al., 2017; Hou et al., 2018). The approved analgesic treatments for CIPN have their undesirable side effects. Gabapentin attenuates pain in CIPN through modulation of voltage-gated calcium channels, transient receptor potential (TRP) ion channels, NMDA receptors, protein kinase C (PKC) inhibition of the membrane, anterograde (axoplasmic) trafficking, inhibition of neurotransmitter release, and inflammation (Kukkar et al., 2013). Microtubule dynamics is not targeted by gabapentin. Thus, gabapentin is likely to ameliorate CIPN symptomatically by affecting nociception, neuropathy being still at large. Therefore, it is imperative to find mechanism-based strategies, without side effects, for preventing and relieving CIPN (Ghoreishi et al., 2018). Fortunately, this concept is evolving fast, albeit with not very promising outcomes yet (Freynhagen and Bennett, 2009; Vardeh et al., 2016; Edwards et al., 2019). PPAR agents like pioglitazone and rosiglitazone have been explored in these regards. These agents are neuroprotective (decreased lesion volume), anti-inflammatory (decreased microglial activation and inflammatory gene expression), anti-apoptotic (decreased number of apoptotic neurons), and antioxidative (Jia et al., 2016). But, weight gain, fluid retention, congestive heart failure, and bone fractures are common adverse effects associated with pioglitazone and rosiglitazone (Nesto et al., 2003; Betteridge, 2011). Among severe side effects and PPAR-γ agonists, rosiglitazone has been associated with increased risks of cardiovascular events, while pioglitazone with significantly increased incidence of bladder cancer (Nissen and Wolski, 2007; Friedland et al., 2012). In 2000, due to hepatotoxicity, troglitazone, yet another PPAR-γ agent, was withdrawn from the clinical use. Dual PPAR-α/γ agonists, like tesaglitazar, muraglitazar, and ragaglitazar have been associated with compound-specific safety, like nonreversible increases in serum creatinine, increase in cardiovascular events, and bladder tumors, respectively; the last one being validated in rodents (Wright et al., 2014). Thus, alternative treatment options in traditional systems of medicines need to be explored.

Indeed, there are reports on efficiencies of extracts from several herbal origins in handling CIPN which has been reviewed by Garg and Adams (2012) and Forouzanfar and Hosseinzadeh (2018). These studies have mostly evaluated single plant extracts and, in few cases, have explored the possible mechanism of action. However, none of these are formulations, thereby limiting their possibility to be used as medication. These studies are very good for enriching the knowledge base on alternative medicinal systems, which, of course, can be used further to identify a successful treatment for CIPN.

Ayurveda, the traditional ancient medicine system of India, relies on protocols and formulations used in combination to treat any ailment. Each treatment regimen is customized based on patient body type with respect to its metabolic efficiency. Ayurvedic medicines are not mere extracts or decoctions. They are well-optimized treatment formulae. Therefore, Ayurvedic treatments aim at eradicating the cause, rather than just subduing or curing the symptoms of the ailment. Divya-Peedantak-Kwath (DPK) is a decoction of 28 herbs, with meticulous optimization with respect to part/s of the plants included, form/s and quantities in which these parts were used (Table 2). We have evaluated its efficacy in treating CIPN in PTX induced neuropathic mouse model. While our observations from behavioral studies convincingly proved that DPK is quite effective in treating CIPN, those from biochemical studies have given insight into the mechanism behind DPK action against CIPN. Our observations indicate that DPK alleviates oxidative stress and prohibits inflammatory response. Besides, its potent antioxidant and anti-inflammatory properties, DPK is able to control PTX-induced weight gain in the animals. However, DPK treatment did not show any other effects on the gross metabolism of the study animals. Weight gain has been reported clinically as a side effect of chemotherapy (Atalay and Küçük, 2015). The animals were monitored throughout the experimental regimen for any clinical sign of toxicity. The absence of toxic side effects is a potential advantage of DPK over gabapentin.

Constituent analysis of DPK revealed its enrichment in polyphenolic compounds, like gallic acid, neochlorogenic acid, cryptochlorogenic acid, vanillic acid, ellagic acid, corilagin, and rutin, which are very effective against oxidative stress, inflammation, and associated immunological responses (Cholet et al., 2019; Jantan et al., 2019). Gallic acid is a very potent antioxidant that can inhibit lipid peroxidation. Its triple phenolic structure helps in metal ion chelation (Badhani et al., 2015). Gallic and ellagic acids can inhibit production of inflammatory cytokines, particularly LPS-induced IL-6. Besides, ellagic acid can normalize the activities of antioxidative enzymes (BenSaad et al., 2017; Chen et al., 2018). Gallic acid is also known to reduce symptoms of inflammation like, pain and swelling (Choubey et al., 2015). Both neochlorogenic and cryptochlorogenic acids suppress inflammation through activation of the AMPK/Nrf2/HO1 pathway (Kim et al., 2015; Gao et al., 2020; Zhao et al., 2020). Vanillic acid can reduce inflammatory pain by inhibiting neutrophil recruitment, oxidative stress, cytokine production, and NF-κB activation (Calixto-Campos et al., 2015). The flavonoid rutin provides neuroprotection through its antioxidant, anti-inflammatory, and immune-modulating capabilities by inhibiting the p38 mitogen–activated protein kinase pathway (Enogieru et al., 2018; Song et al., 2018; da Silva et al., 2020). Corilagin is a potent free radical scavenger that can also inhibit production of pro-inflammatory cytokines and effectors by blocking the NF-κB pathway (Li et al., 2018). Thus, compositional analysis of DPK showed that it is enriched with several antioxidants and anti-inflammatory phytometabolites.

Oxidative stress and inflammation are closely related pathophysiological processes, one of which can be easily induced by another. Thus, both processes are simultaneously found in many pathological conditions, including CIPN (Boyette-davis et al., 2015; Biswas, 2016). Oxidative stress activates a variety of transcription factors involved in inflammatory pathways which can cause many chronic diseases (Hussain et al., 2016). Chemotherapy induces immunomodulatory effects, which in turn causes cytokine-induced neuroinflammation. Neuroinflammation is emerging as one of the major mechanisms of CIPN pathology (Lees et al., 2017). This implicates that herbs with antioxidant, anti-inflammatory, and immunomodulatory properties are likely to be more effective against CIPN. Oxidative stress is known to induce inflammation during CIPN pathogenesis (Areti et al., 2014). Reactive oxygen species (ROS) are implicated in pathophysiology of PTX-induced pain (Duggett et al., 2017). Under oxidative stress, when intra- and extracellular levels of ROS rise in the tissues, the inherent ROS scavenging system of the body employs enzymes like glutathione peroxidases and peroxiredoxins to generate oxidized glutathione (GSSG) from reduced glutathione (GSH). ROS like hydrogen peroxide and organic hydroperoxides get scavenged through reduction during this process (Meister, 1988; Deneke and Fanburg, 1989). Thus, under oxidative stress, tissue levels of GSSG increase. GSH:GSSG serves as an important bioindicator of cellular health. Free radicals generated during oxidative stress lead to lipid peroxidation (Barrera, 2012). Malondialdehyde (MDA) is a biomarker of lipid peroxidation, including when it occurs in the neural tissue as well (Naik et al., 2006; Niki, 2008). We investigated the effect of DPK treatment on oxidative stress in sciatic nerves and that on cytokines levels in human THP-1 cells. DPK treatment reduced the MDA and GSSG levels while increasing GSH:GSSG. The anti-inflammatory effect of DPK was evaluated through its efficiency in reducing the levels of pro-inflammatory cytokines, like IL-6, IL-1β, and TNF-α, in THP-1 cells. LPS was used to induce inflammation in these cells subsequent to their pretreatment with DPK. Besides, serum TNF-α levels were found to be reduced in a dose-dependent manner in response to DPK treatment. Our observations indicate that DPK alleviates oxidative stress and prohibits inflammatory response. *R. communis* (Abdul et al., 2018), *T. chebula* (Bag et al., 2013; Kolla et al., 2018), *T. ammi* (Ranjan et al., 2011; Bairwa et al., 2012), *W. somnifera* (Dar et al., 2016), *A. speciosa* (Joseph et al., 2011), *C. deodara* (Gupta et al., 2011), *V. negundo* (Gill et al., 2018), *N. arbor-tristis* (Hussain and Ramteke, 2012), *T. cordifolia* (Ghosh and Saha, 2012; Khan et al., 2016), *C. fistula* (Kumar et al., 2017; Pawar et al., 2017), *C. sativum* (Mahendra and Bisht, 2011; Rajeshwari and Andallu, 2011), *F. vulgare* (Rahimi and Ardekani, 2013), *Z. officinale* (Shakya, 2015), *S. indicum* (Sharma et al., 2017), and *S. surattense* (Tekuri et al., 2019) are rich in phytochemicals having high antioxidant potentials. *G. herbaceum* (Khaleequr et al., 2012) has antioxidant property, whereas *P. retrofractum* (Kubo et al., 2013) has shown to have neurotrophic effects. Indeed, *T. cordifolia* (Ghosh and Saha, 2012; Khan et al., 2016) and *T. chebula* (Bag et al., 2013; Kolla et al., 2018), in addition to antioxidative property, have anti-inflammatory and immunomodulatory activities. *R. communis* is both an antioxidant and anti-inflammatory agent (Abdul et al., 2018). Besides, being anti-oxidants, *C. deodara* (Gupta et al., 2011) and *Piper longum* (Kumar et al., 2011) are potent immunomodulatory and anti-inflammatory agents. *T. cordifolia* (Khan et al., 2016) and *B. diffusa* (Mahesh et al., 2012) have both anti-inflammatory and immunomodulatory effects. Efficiency of DPK in alleviating oxidative stress can be attributed to these herbal components.

Neuropathic pain is a maladaptive response of the nervous system to damage (Costigan et al., 2009). The peripheral component of the somatosensory nerve senses the pain stimuli and transmits to the central component for processing and response disbursement. A malfunctioning peripheral system due to tissue damage owing to chemotherapy therefore jeopardizes the first step in pain behavior, often leading to over-responsiveness. Restoration of peripheral nerve tissue from the damaging effects of oxidative stress and concomitant inflammation helps in re-establishing appropriate pain responses. Some of the herbal ingredients of DPK have established antinociceptive property, like *N. arbor-tristis* (Hussain and Ramteke, 2012; Kakoti et al., 2013; Sahu and Kharya, 2015), *R. communis* (Abdul et al., 2018), *T. ammi* (Ranjan et al., 2011; Bairwa et al., 2012), *C. zedoaria* (Lobo et al., 2008), and *B. diffusa* (Mahesh et al., 2012; Mishra et al., 2014). Analgesic property of *R. communis* (Abdul et al., 2018), *T. terrestris* (Akram et al., 2011), *P. lanceolate* (Bhagwat et al., 2010; Srivastava and Shanker, 2012), *S. cordifolia* (Galal et al., 2015), *A. speciosa* (Joseph et al., 2011), *N. arbor-tristis* (Kakoti et al., 2013), and *C. zedoaria* (Lobo et al., 2008) is most likely responsible for relieving pain in CIPN. *A. calamus* (Kumar and Vandana, 2013), *T. terrestris* (Akram et al., 2011), and *C. zedoaria* (Lobo et al., 2008), in addition to being analgesic, have anti-inflammatory effects. These observations implicate that DPK is likely to target the oxidative stress–mediated neuroinflammation in neuropathic pain pathology. We observed that DPK treatment alleviated CIPN-associated axonal degeneration and swelling and lesion formation. Lymphocytic infiltration was also reduced. DPK quite efficiently modulated the nociceptive behaviors of the animals with CIPN, implicating a role in re-establishing the tissue structure of peripheral nerves, resulting in reversion of the pain over responses. The assuaging of axonal degeneration, swelling, and lymphocyte infiltration in PTX-induced CIPN can be attributed to the neuroprotective and neurotrophic properties of *W. somnifera* and *P. retrofractum* (Kubo et al., 2013; Dar et al., 2016). In addition, *T. terrestris* serves as a nerve tonic in promoting overall health of the nervous system (Akram et al., 2011). We did not find any ingredient of DPK to directly have effect on microtubule dynamics, a fast-emerging cause of CIPN pathology. However, ROS generated during oxidative stress is known to disrupt microtubule networks, leading to formation of tubulin aggregates in plants (Livanos et al., 2014). So, it is self-evident that attenuating oxidative stress will help restoring the microtubule dynamics. *R. communis* has also been shown to target the peroxisome proliferatoractivated receptor (PPAR) pathway in manifesting its therapeutic effects (Abdul et al., 2018). Since DPK is a collection of active ingredients from several herbs, we cannot exactly attribute its effectivity against CIPN entirely to PPAR-targeting property of *R. communis*. Nevertheless, there are high chances that this pathway is also targeted in addition to others, thereby making DPK highly potent against CIPN.

*R. communis* (Abdul et al., 2018), *T. chebula* (Bag et al., 2013; Kolla et al., 2018), (Kolla et al., 2018), *W. somnifera* (Dar et al., 2016), *V. negundo* (Gill et al., 2018), *C. deodara* (Gupta et al., 2011), *P. longum* (Kumar et al., 2011), *C. fistula* (Kumar et al., 2017), *B. diffusa* (Mahesh et al., 2012), *T. cordifolia* (Ghosh and Saha, 2012), *S. indicum* (Sharma et al., 2017), *P. lanceolate* (Srivastava and Shanker, 2012), and *S. surattense* (Tekuri et al., 2019) have demonstrated anticancer activities mainly due to their antiproliferative and apoptotic effects on cancerous cells. *C. zedoaria* (Lobo et al., 2008) and *C. sativum* (Mahendra and Bisht, 2011; Rajeshwari and Andallu, 2011) have antimutagenic activities. *B. diffusa* has antiestrogenic activity (Mishra et al., 2014), which makes it potentially effective against estrogen-mediated cancers. Chemical analysis of DPK decoction revealed several phytocompounds like gallic, ellagic, vanillic, chlorogenic, cryptochlorogenic, and neochlorogenic acids; corilagin; and rutin. Gallic acid (Badhani et al., 2015), ellagic acid (BenSaad et al., 2017), and rutin (Enogieru et al., 2018) present in these plants impart their anticancer activities. Gallic acid can also modulate cell cycle (Kahkeshani et al., 2019). Chlorogenic acid is known for protecting DNA from mutations (Xu et al., 2012) and corilagin for its apoptotic effect on cancerous cells (Li et al., 2018). Neochlorogenic acid has antitumor activity (Fang et al., 2019). Vanillic acid has antiproliferative activity besides protecting cells from DNA-induced damages (Almeida et al., 2016).

Thus, with such a rich collection of antioxidants, anti-inflammatory agents, and immunomodulatory agents, DPK alleviates neuropathic pain efficiently by targeting the oxidative stress–induced neuroinflammatory pathway. Besides, the anticancer activity associated with the phytocompounds present in DPK provides an added therapeutic advantage against cancer.

## Conclusion

DPK is a combination of several herbs conferring it the ability to target multiple pathways. The constituent herbs of DPK are enriched with phytometabolites with demonstrated antioxidant, anti-inflammatory, immunomodulatory, and neuroprotective activities. Therefore, it is capable of employing a coordinated strike approach against CIPN pathology, by ameliorating the critical causative factors. Moreover, some of the constituent herbs have anticancer property, thereby offering an added advantage against cancer. Taken together, our observations establish DPK as a potent treatment option for CIPN and warrants for further controlled clinical studies.

## Data Availability Statement

The raw data supporting the conclusions of this article will be made available by the authors, without undue reservation, to any qualified researcher.

## Author Contributions

AB and AV conceptualized the study, provided resources, and did formal analysis of the data. AB was responsible for fund acquisition. AV was responsible for project administration, supervision, investigation, data curation and review, and editing of the draft. SS, SK, HS, MT, AK, NS, and PN conducted experiments, analyzed data, and visualized results. SH wrote the original draft. AB and AV finally approved the article.

## Funding

The study received financial and institutional support from the Patanjali Research Foundation Trust founded by Param Pujya Swami Ramdev Ji. The funder played no role in the study design, collection, analysis, interpretation of data, the writing of this article, or the decision to submit it for publication.

## Conflict of Interest

The Patanjali Research Institute (PRI) is an autonomous, nonprofitable organization, governed by the Patanjali Research Foundation Trust, Haridwar, India. PRI is not affiliated with or directed by any commercial interest owned by Patanjali Ayurveda Limited, Haridwar, India. Author AB holds an additional nonprofitable management position in Patanjali Ayurveda Limited, Haridwar, India.

The remaining authors declare that the research was conducted in the absence of any commercial or financial relationships that could be construed as a potential conflict of interest.
